# Coupled Influence of Magnetic Biochar and Solution Chemistries on Retention and Release of Nanoplastics in Porous Media

**DOI:** 10.3390/ijms26052207

**Published:** 2025-02-28

**Authors:** Yan Qin, Yan Liang, Yongtao Peng

**Affiliations:** 1 School of Resources, Environment and Materials, Guangxi University, Nanning 530004, China; 2115303023@st.gxu.edu.cn (Y.Q.);; 2 Guangxi Key Laboratory of Emerging Contaminants Monitoring, Early Warning and Environmental Health Risk Assessment, Nanning 530004, China

**Keywords:** nanoplastics, magnetic biochar, transport, retention, release

## Abstract

Magnetic biochar (MBC), as an environmentally friendly material, has been extensively used for the remediation of soil and groundwater contamination. The retention and release of nanoplastics (NPs) with carboxyl (NPs-COOH) or amino functionalization (NPs- NH_2_) in saturated porous media were investigated under varying conditions of ionic strength (IS), MBC addition, humic acid (HA) concentration, and cation types. The reversible and irreversible retention of NPs was examined by altering the IS, increasing the solution pH, and inducing cation exchange. The results revealed that MBC enhanced the surface roughness of the media, thereby inhibiting NPs’ transport. The HA promoted NPs-NH_2_ transport more effectively than NPs-COOH due to electrostatic repulsion, steric hindrance, and competition for deposition sites. Under a reduced IS and increased pH, a portion of the retained NPs was released, with NPs-NH_2_ showing a greater release than NPs-COOH, indicating reversible retention. Additionally, the stronger charge-shielding and cation-bridging effects of Ca^2+^ significantly enhanced the retention of NPs. Cation exchange resulted in less NPs being released, as most were irreversibly retained in deeper primary minima. However, a small number of retained NPs were remobilized by electrical double layer expansion, surface deprotonation, and cation exchange, indicating reversible retention. These findings provide valuable insights into the fate of NPs in the environment.

## 1. Introduction

With the extensive use of plastic products, plastic pollution has emerged as a pressing global concern. Among its byproducts, nanoplastic particles (NPs) have garnered significant attention due to their inherent biotoxicity and potential ecological risks [1]. Global plastic production exceeded 400 million tons in 2022, with environmental plastic pollution projected to reach 230–370 million tons by 2040 [2]. Soil acts as a major sink for plastics, accumulating them through human activities and posing potential risks to ecosystems [3]. Plastic pollution in soils primarily originates from agricultural irrigation, plastic mulch films, wastewater treatment plant discharges, and atmospheric deposition [4]. In the environment, plastics degrade through aging, weathering, abrasion, and biodegradation, forming primary and secondary particles that further fragment into NPs [5,6], which are defined as plastic particles less than 1000 nm in diameter [7]. NPs are of particular concern due to their high reactivity, which is enhanced by their large surface area that facilitates interactions with contaminants [8,9]. Additionally, their small size increases their mobility, bioavailability, and associated toxicological risks [10,11]. During degradation, NPs acquire various functional groups, which alter their physicochemical properties, such as hydrophobicity and surface charge, and thus affect their fate in subsurface environments [12]. The surface chemical properties of NPs influence their stability and transport. NPs-COOH and NPs-NH_2_ have been frequently used to represent negatively and positively charged NPs, respectively, which exhibit significant differences in behavior and fate in the environment [13]. The transport of NPs in porous media is an essential process that influences their environmental fate in the terrestrial system. Previous studies have revealed that various factors influence the transport of NPs in porous media, e.g., the properties of the porous media, solution chemistries, and the physicochemical properties of the NPs. The surface functional groups of NPs are key determinants of their transport behavior [14,15]. NPs-NH_2_ can adsorb water-soluble polymers, enhancing steric hindrance and increasing repulsive potential energy, thereby promoting their transport in goethite-modified quartz sand. The deposition of NPs-COOH in goethite-coated sand is greater than that of NPs due to an increase in oxygen-containing functional groups which could occur in ligand exchange with Fe atoms in the goethite molecule [12]. In addition, ionic strength (IS), solution pH, cation types, and dissolved organic matter can also significantly influence the environmental behaviors of NPs and other colloidal particles. Increased IS and high-valence cations (such as Ca^2+^ and Al^3+^) tend to promote colloid retention in the primary minimum and deep secondary minimum due to double-layer compression, cation bridging, and charge shielding [16,17]. At a high pH, the deprotonation effect enhances the negative surface charge and the electrostatic repulsion of colloids, thereby facilitating their transport [18]. Humic acid (HA), as a typical dissolved organic matter, has been found to facilitate NP transport by increasing the electrostatic repulsion and steric hindrance between NPs and the collectors [13].

Biochar derived from renewable biomass has long been recognized as a promising adsorbent material and has been widely used as a soil amendment to combat soil pollution [19,20,21]. Magnetic composites can be loaded onto biochar to obtain magnetic adsorbents that can be easily separated. Compared with biochar, magnetic biochar (MBC) has the advantages of easy recycling, no secondary pollution, and high stability [22]. Magnetic biochar demonstrates great potential for the removal of heavy metal ions (e.g., Pb(II), Cd(II), and Cr(VI)) and organic contaminants from aqueous solutions [23,24]. Studies have shown that the incorporation of MBC in quartz sand columns markedly improves the efficiency of plastic particle removal, while simultaneously suppressing their remobilization after a 24 h interruption [25]. Fe_3_O_4_-biochar also inhibits NP transport through mechanisms such as electrostatic adsorption, complexation, and heteroaggregation [26,27]. However, the retention of NPs in porous media is governed by a complex interplay between multiple factors rather than a single determinant. The combined effects of multiple factors, particularly on the transport behavior of NPs with varying surface functional groups in the presence of MBC, remain poorly understood. Additionally, the remobilization of retained NPs under transient solution chemistries has not yet been systematically investigated.

This study aimed to elucidate the transport and retention of NPs (NPs-COOH and NPs-NH_2_) in porous media amended with MBC under varying solution chemistry conditions, including IS, cation types, and HA presence. Batch experiments were conducted to examine the interactions between NPs and MBC, and column transport experiments in conjunction with numerical simulations were used to elucidate the retention mechanisms of NPs. The study further analyzed the differential effects of MBC on the transport and release of NPs with distinct surface functional groups. Additionally, the mechanisms governing the reversibility of retained NPs under transient solution chemistries were explored. The findings provide valuable insights into predicting NP transport, the potential release, and the role of biochar in stabilizing NP retention in the subsurface environment.

## 2. Results and Discussion

### 2.1. Characterization of NPs and MBC

**Figure 1:**
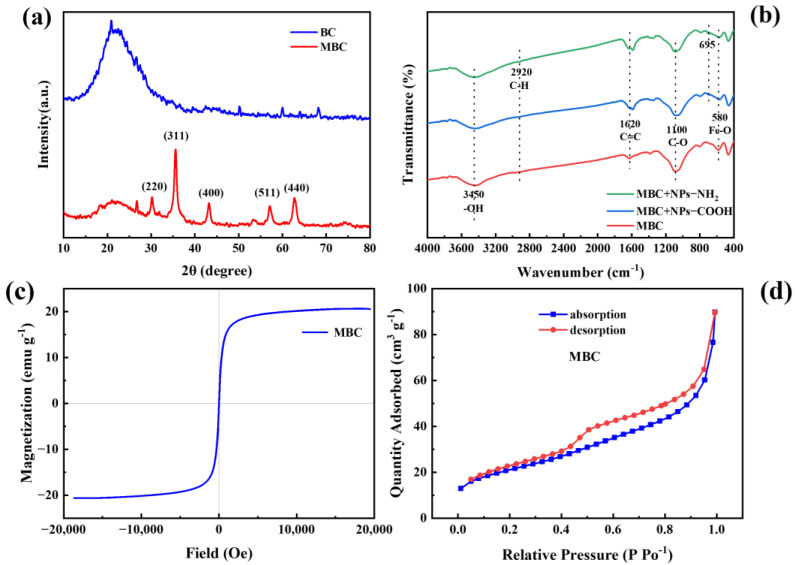
Characteristics of MBC. (**a**) XRD patterns; (**b**) FTIR spectra; (**c**) the vibrating-adsorbent magnetometer of MBC; (**d**) N_2_ adsorption–desorption isotherms of MBC.

**Figure 2:**
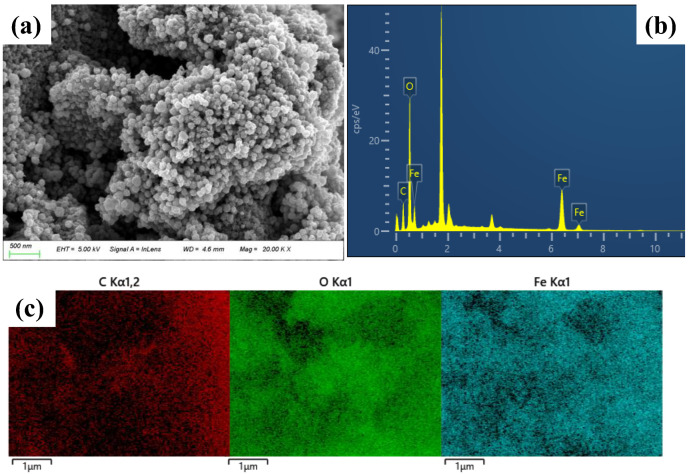
SEM images: (**a**) energy-dispersive X-ray spectrum and (**b**) elemental distribution maps for C, O, and Fe (**c**) in MBC.

The XRD analysis provided insights into the crystal phases of the composite (Figure 1a). The prominent peaks observed in the MBC at 2θ values of approximately 30.24°, 35.65°, 43.23°, 57.07°, and 62.74° corresponded to the (220), (311), (400), (511), and (440) planes of Fe_3_O_4_, respectively [28], confirming the successful synthesis and integration of iron oxide particles onto the biochar’s surface. The FTIR spectra (Figure 1b) further supported this, with a sharp peak at 580 cm^−1^ attributed to Fe-O bonds, indicating the successful loading of metal oxides onto the biochar [29,30,31]. The FTlR spectra demonstrate the presence of carboxyl and amino functional groups on NPs-COOH and NPs-NH_2_, respectively [32]. The hysteresis loop of MBC displays an ‘S’ shape (Figure 1c), indicating excellent superparamagnetic behavior, with a saturation magnetization intensity of 20.64 emu g^−1^. This superparamagnetism facilitates efficient solid–liquid separation and enables the reuse of the adsorbent [33,34]. These results align with the Fe_3_O_4_-related peaks observed in both the XRD and FTIR analyses. The BET-specific surface area of MBC is 75.61 m^2^ g^−1^, suggesting a microporous structure and a rougher surface (Figure 1d and Table S1). The SEM-EDS analysis confirmed the presence of carbon, oxygen, and iron (Figure 2). The atomic percentages are Fe = 15.75%, C = 39.00%, and O = 45.24% for MBC (Table S2), indicating consistent iron loading across the composites.

The zeta potentials and *d_p_* of the NPs and quartz sand under different experimental solution conditions are summarized in Table 1. The zeta potentials became less negative with increasing IS, attributed to double-layer compression. For instance, when the IS increased from 0 mM to 5 mM, the zeta potentials of NPs-COOH, NPs-NH_2_, and the quartz sand shifted from −22.1 ± 0.1 mV, −11.9 ± 2.3 mV, and −26.4 ± 0.3 mV to −9.0 ± 3.2 mV, −7.5 ± 0.5 mV, and −17.4 ± 1.6 mV. In CaCl_2_ solutions, the zeta potentials became even less negative, and *d_p_* increased due to Ca^2+^-induced charge shielding and bridging, promoting NP aggregation [35]. In the presence of 1 mg L^−1^ HA and under 5 mM NaCl, the zeta potentials of NPs-COOH and NPs-NH_2_ were much more negative, with −32.4 ± 0.1 mV and −38.5 ± 1.6 mV, respectively. When the concentration of HA increased from 0 to 1 mg L^−1^ under 5 mM NaCl, the *d_p_* of NPs-COOH was stable, while the *d_p_* of NPs-NH_2_ decreased from 178.1 ± 5.3 nm to 164.8 ± 2.0 nm due to the increased electrostatic and steric repulsion [36]. And, the *d_p_* of NPs-NH_2_ slightly increased from 163.7 ± 1.0 nm to 178.1 ± 5.3 nm when the IS increased from 1 mM to 5 mM.

**Figure 3:**
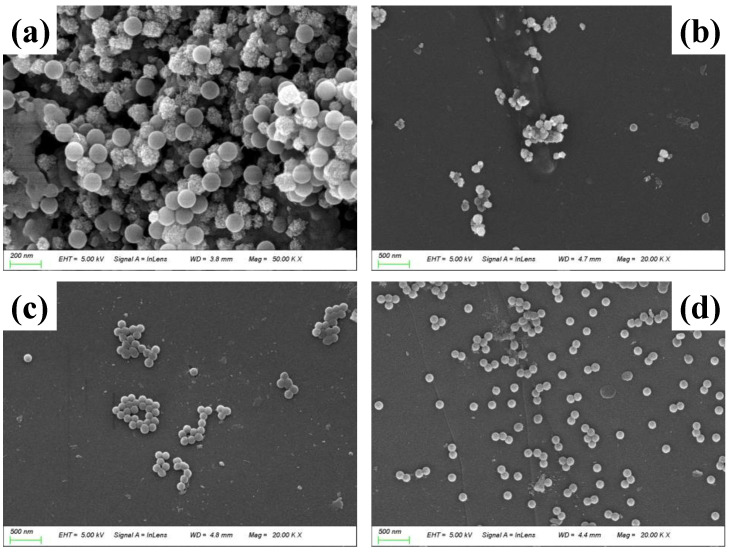
SEM images of NPs. (**a**) NPs adsorbed onto MBC; (**b**) retained NPs on the quartz sand in the presence of MBC; (**c**) retained NPs on the quartz sand; (**d**) retained NPs in the presence of HA.

SEM images (Figure 3) show the attachment of NPs to the MBC. The FTIR spectra peak at 695 cm^−1^ (Figure 1b), corresponding to the stretching vibrations in the C-H benzene ring, and the distinct characteristic peak in the MBC after NP adsorption, confirmed the attachment of NPs onto the MBC’s surface [37]. At a high IS and in CaCl_2_, the NPs were unstable and easily aggregated (Figure 3b). In the presence of MBC, the NPs tended to retain in the region with more MBC (Figure 3c). When HA is present, NPs are more stable and less likely to aggregate (Figure 3d).

### 2.2. Adsorption of NPs on MBC

**Figure 4:**
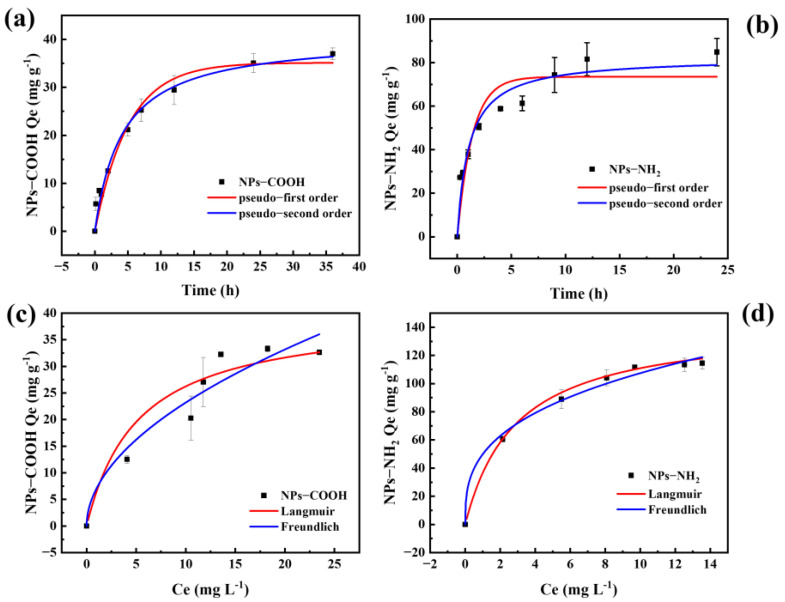
Adsorption kinetics of the MBC for NPs-COOH (**a**) and NPs-NH_2_ (**b**). Adsorption isotherm of the MBC with Langmuir isotherm (red), and Freundlich isotherm (blue) for NPs-COOH (**c**) and NPs-NH_2_ (**d**).

The adsorption kinetics of MBC for NPs (Figure 4a,b) demonstrated a time-dependent increase, with a rapid rise in adsorption capacity within the first 5 h, followed by a gradual deceleration until equilibrium was achieved at 24 h. To elucidate the adsorption mechanism, pseudo-first-order and pseudo-second-order kinetic models were applied. The adsorption kinetics were better described by the pseudo-second-order model (*R*^2^ = 0.980–0.941) compared to the pseudo-first-order model (*R*^2^ = 0.962–0.872) (Table S3). The results suggested that the adsorption process was primarily governed by chemical interactions and the availability of active sites on the MBC’s surface. The predominant mechanism involved chemical adsorption, likely facilitated by the formation of Fe-O-NPs bonds through complexation reactions between MBC and NPs [38].

The adsorption capacity of MBC varied significantly with the charge of the NPs. The less negatively charged NPs-NH_2_ exhibited a notably higher adsorption capacity (*Q_e_* = 82.42 mg g^−1^) and shorter equilibrium time compared to the negatively charged NPs-COOH (*Q_e_* = 40.63 mg g^−1^), which can be attributed to favorable electrostatic interactions [36]. Furthermore, the pseudo-second-order rate constant (*K*_2_) for NPs-NH_2_ (0.013 mg g^−1^ h^−1^) was larger than that of NPs-COOH (0.005 mg g^−1^ h^−1^), reinforcing the observation that NPs-NH_2_ was more readily adsorbed by MBC and reached equilibrium more rapidly. The adsorption isotherms revealed distinct behaviors based on the charge of the NPs. The Freundlich model provided a better fit for NPs-COOH adsorption (*R*^2^ = 0.923), indicating multilayer adsorption on the MBC surface (Figure 4c and Table S3). In contrast, the Langmuir model accurately described the adsorption of NPs-NH_2_ (*R*^2^ = 0.993), suggesting monolayer adsorption (Figure 4d and Table S3) [39,40]. Under identical experimental conditions, the maximum adsorption capacities (*Q_m_*) for NPs-COOH and NPs-NH_2_ were 39.87 mg g^−1^ and 143.13 mg g^−1^, respectively. The higher *Q_m_* and Langmuir constant (*K_l_*) values for NPs-NH_2_ can be attributed to stronger interaction forces, further confirming the preferential adsorption of NPs-NH_2_ by MBC.

### 2.3. Transport and Release of NPs in Saturated Porous Media

**Figure 5:**
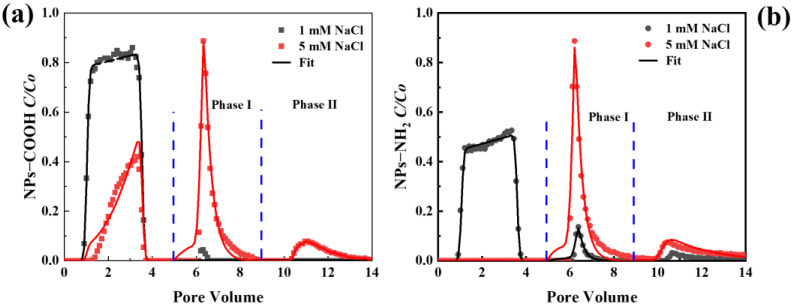
Breakthrough and release curves of NPs-COOH (**a**) and NPs-NH_2_ (**b**) with different ISs at pH 7. The release of NPs was initiated by eluting with ultrapure water under pH 7 (phase I) and pH 10 (phase II), respectively, and the input concentration of NPs was 10 mg L^−1^.

Figure 5 shows breakthrough curves (BTCs) and release curves (RCs) for the NPs with different surface functional groups in the saturated porous media when the solution IS was 1 and 5 mM NaCl at pH 7. The mass recoveries of the NPs are listed in Table S4. As the IS increased from 0 to 5 mM, the transport of NPs decreased, with mass recoveries in the transport phase (*M_eff_*) decreasing from 81.0% and 48.3% to 22.6% and undetectable levels, respectively, as most NPs were retained in the column. This decline in transport was attributed to the reduced electrostatic repulsion and energy barrier with a higher IS, enhancing NP retention [41]. Notably, under identical conditions, NPs-NH_2_ exhibited significantly weaker transport than NPs-COOH, being completely retained in the column at 5 mM IS. The variation in transport between NPs with different functional groups at the same IS can be explained by differences in their zeta potential (Table 1). A higher IS resulted in less negative surface charge for NPs-COOH and NPs-NH_2_, and thus promoted NP retention via reduced energy barriers [42]. In addition, a higher IS also led to the aggregation of NPs-NH_2_ and may lead to surface straining. The fitted transport models effectively described the BTCs, with Pearson correlation coefficients ranging from 0.937 to 0.997 (Table S8). Additionally, the retention parameters *k*_1_ and *S_max_/C* increased with IS, indicating that NP retention was closely related to surface charge heterogeneity [41].

Upon elution with ultrapure water at pH 7 (phase I), a partial release of retained NPs was observed, particularly at 5 mM IS (Figure 5). The recoveries for NPs-COOH and NPs-NH_2_ were 19.2% and 19.8%, respectively (Table S4). This release was attributed to the elimination of the secondary energy minimum or shallow primary minimum [43]. The reduction in IS expanded the electrical double layer, facilitating NP remobilization. During phase II elution with ultrapure water under pH 10, additional release of NPs-COOH (9.2%) and NPs-NH_2_ (10.6%) occurred due to surface deprotonation and increased electrostatic repulsion initiated by the more negative surface charge [44]. However, substantial fractions of the NPs remained in the column (49% of NPs-COOH and 69.5% of NPs-NH_2_), suggesting that these particles were trapped within the deep primary minimum [45]. This highlights the strong retention capacity of porous media under high IS conditions, particularly for NPs-NH_2_.

### 2.4. Effect of MBC on Transport and Release of NPs

**Figure 6:**
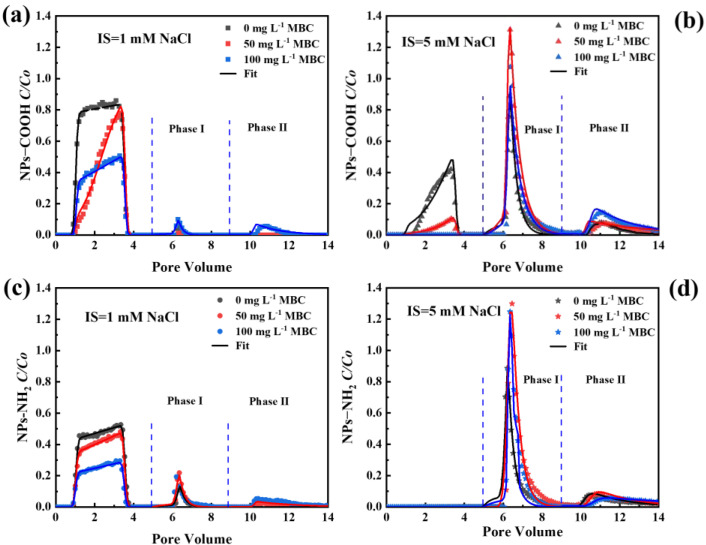
Breakthrough and release curves of NPs-COOH in 1 (**a**) and 5 (**b**) mM NaCl, and breakthrough and release curves of NPs-NH_2_ in 1 (**c**) and 5 (**d**) mM NaCl at different concentrations of MBC. The release of NPs was initiated by eluting with ultrapure water under pH 7 (phase I) and pH 10 (phase II), respectively, and the input concentration of NPs was 10 mg L^−1^.

Figure 6 shows the transport and release of NPs under 1 or 5 mM NaCl at different MBC concentrations. The mass recoveries of NPs under different experimental conditions are shown in Table S5. Experimental and numerical simulation results indicated that MBC and IS significantly influenced the transport of NPs (Table S8). Under 1 mM NaCl, the *M_eff_* of NPs-COOH and NPs-NH_2_ decreased from 81.0% and 48.3% to 42.5% and 25.4%, when the concentration of MBC increased from 0 to 100 mg L^−1^. These results suggested that the MBC significantly inhibited the mobility of NPs within the columns. The fitted values of *k*_1_ and *S_max_/C_o_* increased with the concentration of MBC, indicating increased retention capacities. Under 5 mM NaCl, NPs-NH_2_ was almost completely retained in the column. It should be noted that there were negligible changes in column porosity in the presence of MBC. Adsorption experiments demonstrated that MBC exhibited a strong affinity for NPs, with the maximum adsorption capacity (*Q_m_*) reaching 39.89 mg g^−1^ for NPs-COOH and 143.13 mg g^−1^ for NPs-NH_2_ (Table S3). In contrast, a previous study reported that quartz sand had a negligible adsorption capacity for NPs, with only 0.16 mg g^−1^ of NPs being adsorbed [32]. The oxygenated functional group (-COOH) on NPs-COOH could bind with Fe-O on MBC to form a COO-(FeO) ligand complex, which promotes the adsorption of NPs-COOH by MBC [46]. The SEM images of the interactions between NPs and MBC demonstrate that more NPs were binding to the MBC (Figure 3b).

Additionally, adding MBC to quartz sand could increase the roughness of the collector surface. The SEM image in Figure 3b reveals that the surface of quartz sand exhibited more convex sites due to the attachment of MBC. According to the N_2_-BET analysis, MBC had a larger specific surface area (75.61 m^2^ g^−1^) compared to quartz sand (1.006 m^2^ g^−1^) [47], thereby enhancing the availability of favorable retention sites for NPs. Surface morphology can reduce or eliminate energy barriers, promoting the formation of a shallow primary minimum [48]. Therefore, with the addition of MBC, the energy barrier between the NPs and the porous media was lowered, enhancing NP retention. Additionally, as shown in Figure 2a, the MBC exhibited a varied morphology with a developed pore structure, allowing NPs to be trapped in the concave areas, further improving retention.

The RCs in Figure 6 show that at an IS of 5 mM NaCl, a significant proportion of the NPs (*M_I_* = 19.3–35.3%) was released from the porous media during the pH 7 ultrapure water elution phase. This indicated that some NPs were retained in the secondary or shallow primary minimum, allowing for reversible retention. In phase II (elution with ultrapure water at pH 10), only a small fraction of the NPs was released due to surface deprotonation. A large fraction (51.7–63.7%) remained retained in the columns in the presence of MBC. This suggests that most NPs were irreversibly trapped in the deep primary minimum, making remobilization difficult even under transient solution chemical conditions. At a concentration of 100 mg L^−1^ MBC and 5 mM IS, 27.4% of the NPs-NH_2_ retained in the packed column was released from the porous media during phase I. In comparison, 25.5% of the NPs-COOH was released under identical conditions. The higher *M_I_* value for NPs-NH_2_ was primarily attributed to greater retention during the deposition phase. In contrast, during phase II, NPs-COOH (20.1%) were released in greater quantities than NPs-NH_2_ (11.4%), suggesting that the interactions between NPs-NH_2_ and the sand were stronger than those between NPs-COOH and the sand.

### 2.5. Effect of HA on Transport and Release of NPs

**Figure 7:**
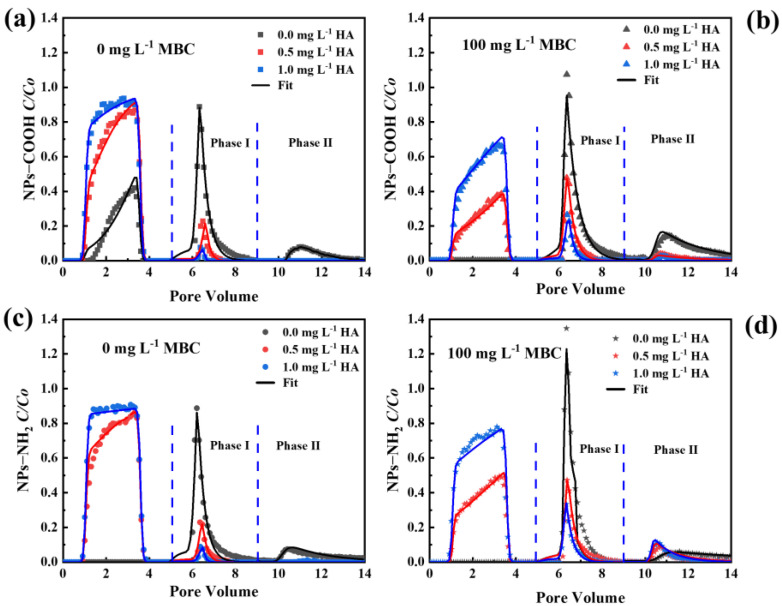
Breakthrough and release curves of NPs-COOH in the absence (**a**) and presence (**b**) of MBC, and breakthrough and release curves of NPs-NH_2_ in the absence (**c**) and presence (**d**) of MBC at different HA concentrations. The release of NPs was initiated by eluting with ultrapure water under pH 7 (phase I) and pH 10 (phase II), respectively; the input concentration of NPs was 10 mg L^−1^ and the IS was 5 mM NaCl.

The BTCs and RCs of NPs-COOH and NPs-NH_2_ with varying HA concentrations without and with MBC presence (0, 0.5, and 1.0 mg L^−1^) under an IS of 5 mM NaCl are shown in Figure 7. The presence of HA in NP suspensions significantly promoted NP transport compared to its absence. In the absence of MBC, as the HA concentration increased from 0 to 1 mg L^−1^, the *M_eff_* values of NPs-COOH and NPs-NH_2_ increased from 22.6% and <0.01% to 86.7% and 87.3%, respectively. In the presence of MBC, the *M_eff_* values for NPs-COOH and NPs-NH_2_ increased from <0.01% to 53.9% and 68.6% with increasing HA, respectively (Table S6). The fitted values of *k_1_* and *S_max_/C_o_* decreased with increasing HA concentration (Table S8), further indicating that HA reduced the retention capacities for NPs. This was due to the increased electrostatic and steric repulsion between the NPs and collectors [49].

The comparisons in Figure 7 reveal that HA significantly differed in the transport of NPs with different functional groups. In the presence of HA, the zeta potential of NPs-NH_2_ became more negative than that of NPs-COOH (Table 1). Therefore, the HA promoted the transport of NPs-NH_2_ more than NPs-COOH because stronger the repulsion against the collector surface when the HA neutralized the portion of positive charge on NPs-NH_2_ [50]. Additionally, the surface roughness of NPs-COOH may increase due to the formation of complexes with HA [15]. These alterations will lead to a greater mobility for NPs-NH_2_ than NPs-COOH.

In the presence of HA without MBC (Figure 7a,c), about 0.7–3.9% of NPs were released under elution with ultrapure water at pH 7 (phase I), indicating reversible NP retention. However, no NP release was observed at an increased solution pH (phase II), suggesting that NPs were retained in the deep primary minimum. In the presence of MBC and HA, 4.1–10.1% of the NPs were released in phase I, while 0.9–6.0% were released in phase II (Table S6), indicating that some NPs were retained in the shallow primary minimum in a reversible form. However, at a concentration of 1 mg L^−1^ HA, approximately 18–40% of the NPs remained irreversibly retained in the sand column, which was lower than in the absence of HA. This result indicates that the presence of HA reduced the retention of NPs in the column. This reduction may be due to stronger electrostatic and steric repulsion between the NPs and quartz sand in the presence of HA. Additionally, the Fe on the MBC could form complexes with HA, and the competition for deposition sites by HA facilitated the transport of NPs through the porous media [26,51]. Furthermore, the surface functional groups of the NPs also influence their release, with a greater release of NPs-NH_2_ during both phase I and phase II compared to NPs-COOH. Under the conditions of 100 mg L^−1^ MBC and 1 mg L^−1^ HA, 6.5% of NPs-NH_2_ was released from the porous media in phase I and 6.0% in phase II, whereas only 4.1% of NPs-COOH was released in phase I and 0.9% in phase II.

### 2.6. Effect of Divalent Cations on the Transport and Release of NPs

**Figure 8:**
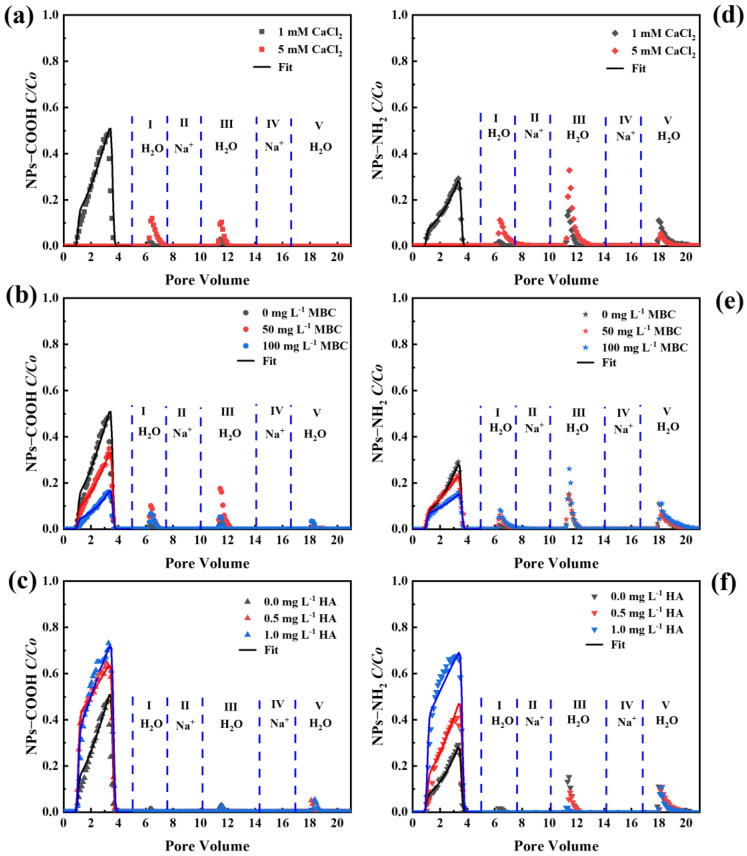
Breakthrough and release curves of NPs-COOH (**a**–**c**) and NPs-NH_2_ (**d**–**f**) in CaCl_2_: (**a**,**d**) under different IS; (**b**,**e**) at different concentrations of MBC and absence of HA under 1 mM CaCl_2_; (**c**,**f**) at different concentrations of HA and absence of MBC under 1 mM CaCl_2_. Released NP was initiated by eluting with H_2_O, 1 mM NaCl, H_2_O, 100 mM NaCl, and H_2_O in release phases I–V, respectively.

The effects of divalent cations on the transport and release of NPs in porous media are illustrated in Figure 8. Compared to Na^+^, Ca^2+^ exhibited a stronger inhibitory effect on NP transport under the same IS conditions. In CaCl_2_ solution, the zeta potentials of both the NPs and quartz sand were much less negative than those in NaCl, implying stronger charge neutralization. When the ionic strength (IS) was 5 mM, NPs-COOH were completely retained in the column, resulting in a *M_eff_* value of less than 0.01 in the CaCl_2_ solution. In contrast, the *M_eff_* value of NPs-COOH was 22.6% in the NaCl solution. As a result, the retention of NPs was more pronounced in Ca^2+^ (Table S7) due to cation bridging and a reduced energy barrier [16]. The fitted values of *S_max_*/*C_o_* were higher in CaCl_2_ than in NaCl (Table S9), indicating increased retention capacities.

The inhibitory effect of Ca^2+^ on NP transport was more pronounced in the presence of MBC, which increased the surface roughness and specific surface area, thus decreasing the energy barrier and increasing retention sites [52]. Furthermore, as the HA concentration increased, NP transport was again enhanced due to the increased energy barrier. Under the same IS, NPs-COOH exhibited larger *d_p_* (Table 1), indicating larger aggregation, primarily due to the carboxyl groups on NPs binding more easily to Ca^2+^ [53]. In contrast, the transport of NPs-NH_2_ was weaker due to weaker repulsions under the same conditions. In the Ca^2+^ solution, HA promoted the transport of NPs-COOH more effectively than in the Na^+^ solution. This was attributed to the interaction between Ca^2+^ and HA, which led to cation-π adsorption of HA-Ca complexes on NPs-COOH [54]. Therefore, the electrostatic repulsion was stronger than the charge screening effect.

The release of NPs under cation exchange and reduced IS conditions was studied by sequential elution of packed columns with H_2_O, 1 mM Na^+^, H_2_O, 100 mM Na^+^, and H_2_O. A small release of NPs (0.2–2.4%) (Table S7) occurred during IS reduction (phase I) compared to Na^+^, indicating that the retained NPs were trapped in the deep primary minimum in the presence of Ca^2+^. When MBC was present (Figure 8b,e), a small portion of NPs was released during phase I (0.2–2.4%) and phase III (0.4–3.5%). The release of NPs-NH_2_ was more pronounced compared to NPs-COOH. However, no NP release was observed during phase I in the presence of HA (Figure 8c,f). The cation-bridging effect of Ca^2+^ led to irreversible retention in the primary minimum when IS was reduced [55]. A small fraction of NPs-NH_2_ (0.2–3.6%) was released during phase V. Elution with 100 mM Na^+^ during phase IV enhanced the repulsive hydration force, initiating cation exchange, which diminished the cation bridging and reduced charge heterogeneity from adsorbed Ca^2+^ [56]. These factors strengthened the electrostatic repulsion and promoted NP release in phase V, when the column was eluted with ultrapure water (expansion of the double layer). This finding indicated that while a small number of NPs can be released through cation exchange and IS reduction, the majority undergo irreversible retention, being deposited in the deep primary minimum where they cannot be remobilized under transient solution chemistry conditions. The RCs show an increased release of NPs-NH_2_ after cation exchange, likely because these fractions of NPs-NH_2_ were retained in the shallower primary minimum, thus they could be remobilized upon cation exchange and IS reduction.

## 3. Materials and Methods

### 3.1. NPs, MBC, Solution Chemistries and Porous Media

Spherical polystyrene nanoplastics (NPs) (Suzhou Mylife New Material Technology Co., Ltd., Suzhou, China) with an average size of approximately 100 nm were employed as model NPs. To examine the influence of surface functional groups on the transport and release of NPs in saturated porous media, NPs functionalized with carboxyl groups (NPs-COOH) and amino groups (NPs-NH_2_) were selected. A high-concentration NP suspension (1 g L^−1^) was diluted with NaCl or CaCl_2_ background solutions to a final concentration of 10 mg L^−1^ for use as the influent in column experiments. The NP concentrations in the suspension were quantified using a fluorescence spectrophotometer (F-320, Tianjin GD Technology, Tianjin, China) with excitation and emission wavelengths of 350 nm and 666 nm, respectively, calibrated using a standard curve. The zeta potential and hydrodynamic diameter of NPs under different solution chemistry conditions (1 mM and 5 mM Na^+^ or Ca^2+^, and varying concentrations of HA) were measured using a Zetasizer (Nano ZS90, Malvern Instruments, Worcestershire, UK). The retention of NPs on collector surfaces was visually characterized using scanning electron microscopy (SEM, ZEISS Sigma 300, Neustadt, Germany).

The biochar used in this study was produced by pyrolyzing corn stover (sourced from Lianyungang, Jiangsu Province) in a nitrogen atmosphere at 800 °C for 2 h in a tube furnace. The MBC was synthesized via a hydrothermal reaction in an organic solvent, with reference to a previous study [57]. In brief, 2 g of biochar, 1.6 g of FeCl_6_·H_2_O, and 3.2 g of CH_3_COONa were added to 70 mL of ethylene glycol solvent, mechanically stirred for 30 min, and subsequently sonicated for another 30 min. The mixture was then transferred to a stainless-steel high-pressure reactor and heated to 230 °C for 24 h. Following the reaction, the composites were magnetically separated, washed repeatedly with ethanol and ultrapure water until the supernatant was clear, and subsequently vacuum freeze-dried for storage. Surface morphology of MBC was characterized by scanning electron microscopy (SEM, ZEISS Sigma 300, Germany). The elemental composition was characterized by the SEM with energy-dispersive X-ray spectroscopy (EDS, Xplore compact, Oxford Instruments, Oxford, UK). The crystal structures of BC and MBC were qualitatively analyzed using an X-ray diffractometer (XRD, D8 Discover, Bruker, Berlin, Germany) with diffraction angles ranging from 20 to 80° and a sampling time of 10°/min. Fourier transform infrared spectroscopy (FTIR, Shimadzu, Kyoto, Japan) was used to determine the functional groups in the 400–4000 cm^−1^ range. A vibrating sample magnetometer (VSM, Lakeshore-7410, Columbus, OH, USA) was used to characterize the magnetization intensity of the MBC with a magnetic field range of ±2 T. Specific surface area and pore structure were assessed by N_2_ adsorption/desorption isotherms derived from an automated specific surface area analyzer (ASAP2460, McMurtic Instruments, Atlanta, GA, USA).

Electrolyte background solutions (1 mM and 5 mM NaCl or CaCl_2_) were prepared by diluting stock solutions (100 mM) with ultrapure water (Milli-Q, Darmstadt, Germany) and adjusted to pH 7 using NaOH. For release experiments, ultrapure water adjusted to pH 7 or 10 was used as the eluent. Humic acid (HA; Sigma-Aldrich, Shanghai, China) was used as a model for dissolved organic matter. HA powder was used to formulate a highly concentrated stock solution. Then, stock HA suspensions (100 mg L^−1^) were diluted into the background electrolyte solutions containing NPs (10 mg L^−1^) to achieve final HA concentrations of 0.5 mg L^−1^ and 1 mg L^−1^.

Quartz sand (purchased from Tianjin Guangfu Fine Chemical Research Institute, Tianjin, China) was used as the porous medium in the column transport experiments. The sand was purified to remove surface contaminants, including metal oxides and organic matter. First, the sand was rinsed with tap water to remove dust and debris. To eliminate metal oxides, the sand was soaked in 65% HNO_3_ for 24 h, followed by thorough rinsing with water until the pH stabilized at approximately 7. Organic matter was then removed by immersing the sand in 10% H_2_O_2_ for 24 h, followed by repeated water rinsing. To eliminate colloidal impurities and expand the double layer, the sand was treated sequentially with a high ionic strength electrolyte solution (100 mM NaCl) and ultrapure water at pH 10 for 2 h. After ultrasonication for 2 h, the sand was oven-dried at 50 °C and sieved through a 40–60 mesh sieve for use in experiments.

### 3.2. Batch Experiments

A series of batch experiments were conducted to evaluate the adsorption of NPs onto MBC. For the kinetic studies, 10 mg MBC was ultrasonicated for 15 min to ensure uniform dispersion of the adsorbent and subsequently mixed with NPs. The suspensions (50 mL 100 mg L^−1^ MBC and 20 mg L^−1^ NPs) were shaken in a water bath shaker at 180 rpm and maintained at 25 °C. Samples were collected at predetermined intervals (0.5, 1, 2, 4, 6, 9, 12, and 24 h). To separate the MBC from the mixture, a strong magnet was placed close to the bottom of the conical flask containing the mixture, and the MBC was attracted by a magnetic field and thus removed. The concentrations of NPs in the supernatants were analyzed using a fluorescence spectrophotometer to determine the time for adsorption equilibrium. For the adsorption isotherm experiments, varying initial concentrations of NPs (20, 25, 30, 35, and 40 mg L^−1^) were combined with 10 mg of MBC and shaken for 24 h under identical conditions (180 rpm, 25 °C). After the separation of MBC using magnets, the residual NP concentrations in the supernatants were quantified via fluorescence spectrophotometry. All experiments were conducted in triplicate with one blank sample included for each condition to ensure the accuracy and reliability of the results.

### 3.3. Column Experiments

#### 3.3.1. Transport Experiments

The columns (12 cm in length and 3 cm in internal diameter) were wet-packed with quartz sand and were employed under water-saturated conditions. Column porosity was determined using a gravimetric method by comparing the weights of the saturated column, the empty column, and the packed sand. A peristaltic pump (HL-1D, Shanghai Huxi, Shanghai, China) maintained a consistent upward flow of the background solution, tracer, MBC suspension, and nanoparticle (NP) suspension at a constant Darcy velocity of 0.7 cm min^−1^. Prior to transport experiments, the columns were pre-conditioned by elution with approximately 50 pore volumes (PVs) of background solution with ionic strengths (IS) of 1 or 5 mM NaCl or CaCl_2_ at pH 7. Afterward, the transport of the tracer was examined by injecting 100 mL of NaNO_3_ or Ca(NO_3_)_2_ solution with the same IS and pH as the background solution, followed by elution with around 100 mL of the background solution. Subsequent NP transport experiments involved injecting 100 mL of NP suspension into the column, followed by elution with 100 mL of NP-free background solution. Effluent samples from both tracer and NP transport experiments were collected using a fraction collector (CBS-A, Shanghai Huxi, China). Tracer concentrations were determined spectrophotometrically using a UV spectrophotometer (UV-2450, Shimadzu, Japan) at a wavelength of 235 nm, while NP concentrations were quantified using a fluorescence spectrophotometer to obtain breakthrough curves (BTCs) [32].

In the transport experiment involving MBC, following the tracer test, 100 mL of MBC suspension at selected concentrations (0, 50, and 100 mg L^−1^) was injected into the column. The mass ratio of MBC to porous media was less than 0, 0.004%, and 0.008%, respectively. This was followed by elution with 20 PVs of background solution until no detectable MBC remained in the effluent. Subsequently, the NP transport experiment was conducted under identical conditions and procedures as previously described.

#### 3.3.2. Release Experiments

Release experiments were performed following the NP transport experiments to evaluate the reversibility of NP retention under varying solution conditions, including reductions in ionic strength (IS), increases in pH, and cation exchange. NP release was investigated in two phases when the background solution contained 1 or 5 mM NaCl. After completing the transport experiments, the columns were flushed with 160 mL of ultrapure water at pH 7 (phase I) and 200 mL of ultrapure water at pH 10 (phase II) to examine the remobilization of retained NPs from primary or secondary potential wells. With CaCl_2_ as the background solution, NP release was studied across five phases to assess retention reversibility under conditions of cation exchange and IS reduction. The detailed protocol included sequential flushing of the column with the following solutions: 100 mL of ultrapure water at pH 7 (phase I), 100 mL of 1 mM NaCl (phase II), 150 mL of ultrapure water at pH 7 (phase III), 100 mL of 100 mM NaCl (phase IV), and 200 mL of ultrapure water at pH 7 (phase V). The NP concentrations in the effluent were measured to obtain release curves (RCs).

### 3.4. Adsorption and Transport Models

The pseudo-first-order and pseudo-second-order kinetic models were applied to evaluate the adsorption kinetics of MBC with NPs. Adsorption isotherms were characterized using the Langmuir and Freundlich models. The adsorption rate and capacity of MBC were determined based on the kinetic and isotherm data derived under experimental conditions. Additional details regarding the model simulations are available in Supplementary Materials.

The convection–dispersion equation (CDE) was employed to describe NP transport within the liquid phase and from the liquid to the solid phase. Parameters such as dispersity (*D*), porosity (*θ*), and bulk density (*ρ*) were obtained through fitting to the tracer BTCs. The retention rate coefficient (*k*_1_) and the normalized maximum solid-phase concentration (*S_max_/C*_0_) were obtained by fitting to the BTCs of NPs using HYDRUS-1D. Further details can be found in Supplementary Materials.

## 4. Conclusions

This study examines the coupled roles of MBC, HA, and cation types on the retention and release of nanoplastics (NPs) with different surface functional groups (-COOH and -NH_2_). Increasing IS inhibited NP transport by compressing the electrical double layer, while MBC enhanced surface roughness and created additional deposition sites, promoting NP retention. NPs-NH_2_ were more sensitive to IS and MBC. The HA facilitated NP transport via increased steric repulsion and a surface complex with a stronger effect on NPs-NH_2_ than NPs-COOH due to electrostatic interactions. Under transient conditions (reduced IS and increased pH), partial remobilization of NPs occurred from the shallow primary minima. The release of NPs-NH_2_ was more pronounced than that of NPs-COOH due to previous greater retention with reversible interactions. In Ca^2+^ solutions, NP transport was more inhibited than in Na^+^ solutions due to cation bridging and charge shielding. The HA promoted NPs-COOH transport more effectively in Ca^2+^, though only a small fraction of the retained NPs were remobilized, indicating irreversible retention in deeper minima. When the particles were retained in the presence of a divalent cation, more NPs-NH_2_ was released compared to NPs-COOH, but still less than that under monovalent cation conditions due to stronger irreversible interactions. This study reveals that a limited amount of MBC can effectively enhance the irreversible retention of NPs in subsurface environments and demonstrates the remobilization of NPs under transient solution chemical conditions. Future work should focus on better understanding the fate of NPs in natural soils, different species of NPs, and pollutants coexisting with NPs.

## Figures and Tables

**Table 1:** Zeta potentials of NPs and quartz sand, and the hydrodynamic diameters (*d_p_*) of NPs under different experimental conditions.

Sample	Cation Types	IS	HA (mg L^−1^)	pH	*d_p_*, nm	Zeta Potential, mV
NPs-COOH	0	0	0	7	156.9 ± 1.7	−22.1 ± 0.1
	0	0	0	10	187.3 ± 0.9	−28.2 ± 0.3
	NaCl	1	0	7	169.9 ± 0.9	−17.2 ± 1.7
	NaCl	5	0	7	171.0 ± 1.3	−9.0 ± 3.2
	NaCl	5	0.5	7	170.8 ± 5.4	−14.3 ± 2.1
	NaCl	5	1	7	170.7 ± 1.6	−32.4 ± 0.1
	CaCl_2_	1	0	7	171.2 ± 2.7	−6.0 ± 4.4
	CaCl_2_	1	0.5	7	170.6 ± 1.1	−4.5 ± 0.9
	CaCl_2_	1	1	7	169.5 ± 5.5	−6.6 ± 0.9
	CaCl_2_	5	0	7	215.7 ± 3.2	−3.6 ± 3.9
NPs-NH_2_	0	0	0	7	162.5 ± 1.0	−11.9 ± 2.3
	0	0	0	10	156.8 ± 5.7	−26.0 ± 0.9
	NaCl	1	0	7	163.7 ± 1.0	−16.7 ± 1.5
	NaCl	5	0	7	178.1 ± 5.3	−7.5 ± 0.5
	NaCl	5	0.5	7	166.8 ± 2.1	−30.7 ± 1.6
	NaCl	5	1	7	164.8 ± 2.0	−38.5 ± 1.6
	CaCl_2_	1	0	7	162.2 ± 0.8	−7.0 ± 3.7
	CaCl_2_	1	0.5	7	161.5 ± 0.1	−14.1 ± 2.4
	CaCl_2_	1	1	7	161.2 ± 2.4	−20.2 ± 0.3
	CaCl_2_	5	0	7	178.5 ± 3.0	−5.2 ± 2.2
Sand	0	0	0	7	-	−26.4 ± 0.3
	0	0	0	10	-	−46.8 ± 3.7
	NaCl	1	0	7	-	−35.2 ± 0.6
	NaCl	5	0	7	-	−17.4 ± 1.6
	NaCl	5	0.5	7	-	−36.3 ± 5.3
	NaCl	5	1	7	-	−37.1 ± 1.4
	CaCl_2_	1	0	7	-	−20.6 ± 0.7
	CaCl_2_	1	0.5	7	-	−26.0 ± 0.2
	CaCl_2_	1	1	7	-	−29.4 ± 1.5
	CaCl_2_	5	0	7	-	−15.9 ± 3.0

Note: “-” indicates no detection.

## Data Availability

The data that support the findings of this study are available from the corresponding author, Y.L., upon reasonable request.

## Supplementary Materials

The following supporting information can be downloaded at: https://www.mdpi.com/article/10.3390/ijms26052207/s1. References [58,59,60,61] are cited in the Supplementary Materials.
