# Supplementary material for: Coupled Influence of Magnetic Biochar and Solution Chemistries on Retention and Release of Nanoplastics in Porous Media
Source: Int J Mol Sci. 2025 Feb 28;26(5):2207. doi: 10.3390/ijms26052207 (PMC11899741; doi:10.3390/ijms26052207)
Supplement: Supplementary file 1 [file ijms-26-02207-s001.zip › ijms-3495711-supplementary.pdf]

Supplementary Materials

Coupled Influence of Magnetic Biochar and Solution Chemistries on Retention and Release of Nanoplastics in Porous Media

Yan Qin [1,2], Yan Liang [1,2,*] and Yongtao Peng [1,2]

[1] *School of Resources, Environment and Materials, Guangxi University, Nanning 530004, China*

[2] *Guangxi Key Laboratory of Emerging Contaminants Monitoring, Early Warning and Environmental Health Risk Assessment, Nanning 530004, China*

* Correspondence: liangyan@gxu.edu.cn

Introduction

Supplementary information includes a brief description and discussion of the following: (i) Adsorption models (S1); (ii) Transport model (S2); (iii) The N_2 BET specific surface areas of MBC (Table S1); (iv) Relative atomic percentages of major elements on the MBC surface analyzed by EDS (Table S2); (v) Adsorption kinetics and isotherm parameters for NPs adsorption by MBC (Table S3); (vi) The mass recoveries of NPs at different experimental conditions under NaCl solution (Table S4); (vii) The mass recoveries of NPs at different experimental conditions under NaCl solution (Table S5); (viii) The mass recoveries of NPs at different experimental conditions under NaCl solution (Table S6); (ix) The mass recoveries of NPs at different experimental conditions under $CaCl_2$ solution (Table S7); (x) Fitted values of NPs transport at different experimental conditions under NaCl solution (Table S8); (xi) Fitted values of NPs transport at different experimental conditions under $CaCl_2$ solution (Table S9).

Section S1. Adsorption models

In this study, pseudo-first-order (Eq. S1) and pseudo-second-order (Eq. S2) kinetic models were used to fit the adsorption kinetic of MBC on NPs [1–3].

Pseudo-first-order kinetic equation:

$$Q_t = Q_e(1 - e^{-k_1 t}) \tag{S1}$$

Pseudo-first-order kinetic equation:

$$Q_t = \frac{Q_e^2 k_2 t}{Q_e k_2 t + 1} \tag{S2}$$

where K_1 (h^{-1}), pseudo-first-order kinetic rate constant; K_2 (mg g^{-1} h^{-1}), pseudo-second-order kinetic rate constant; Q_e, (mg g^{-1}), equilibrium adsorption capacity.

Langmuir (Eq. S3) and Freundlich (Eq. S4) models were employed to describe the adsorption isotherms [3].

Langmuir equation:

$$Q_e = \frac{K_L Q_m C_e}{1 + K_L C_e} \tag{S3}$$

Freundlich equation:

$$Q_e = K_F C_e^{1/n} \tag{S4}$$

where Q_m (mg g^{-1}), maximum adsorption; K_L (L mg^{-1}), distribution coefficient; K_F (L mg^{-1}), Freundlich constant; n, the favorability degree of the adsorption process.

Section S2. Transport models

Transport of NPs in the aqueous phase was described using the advection-dispersion equation (CDE) that includes an exchange term to/from the aqueous and the solid phases:

$$\frac{\partial(\theta_w C)}{\partial_t} + \frac{\partial(\rho_b S)}{\partial_t} = \frac{\partial}{\partial_z}\left(\theta_w D \frac{\partial_c}{\partial_z}\right) - \frac{\partial(qC)}{\partial_z} \quad \text{(S5)}$$

where C [NL^{-3}; where N and L denote units of number and length, respectively] is the NPs concentration in the aqueous phase, D [L^2 T^{-1}; where T denotes units of time] is the hydrodynamic dispersion coefficient for NPs, θ_w [L^3 L^{-3}] is the volumetric water content, ρ [M L^{-3}; where M denotes units of mass] is the soil bulk density, and S [N M^{-1}] is the solid phase NPs concentration. The first and second terms on the right-hand side of Eq. (S5) account for the dispersive and advective fluxes of NPs, respectively.

The solid phase mass balance equation during steady-state solution chemistry conditions is given in this work as:

$$\frac{\partial(\rho_b S)}{\partial_t} = \theta_w k_1 \psi C \quad \text{(S6)}$$

where k_1 [T-1] is the retention rate coefficient, and ψ [-] is the Langmuirian blocking function.

The Langmuirian blocking function [4] as:

$$\psi = \frac{S_{max} - S}{S_{max}} \quad \text{(S7)}$$

where S_{max} [N M^{-1}] is the maximum solid phase NPs concentration.

References

1. Ho, Y. The kinetics of sorption of divalent metal ions onto sphagnum moss peat. *Water Res.* **2000**, *34*, 735–742. https://doi.org/10.1016/s0043-1354(99)00232-8.
2. Ho, Y.S.; McKay, G. Kinetic models for the sorption of dye from aqueous solution by wood. *Process Saf. Environ. Prot.* **1998**, *76*, 183–191. https://doi.org/10.1205/095758298529326.
3. Langmuir, I. The adsorption of gases on plane surfaces of glass, mica and platinum. *J. Am. Chem. Soc.* **1918**, *40*, 1361–1403. https://doi.org/10.1021/ja02242a004.
4. Adamczyk, Z.; Siwek, B.; Zembala, M.; Belouschek, P. Kinetics of localized adsorption of colloid particles. *Adv. Colloid Interface Sci.* **1994**, *48*, 151–280. https://doi.org/10.1016/0001-8686(94)80008-1.

Table S1 The N_2-BET specific surface area of MBC

N_2-BET ($m^2\ g^{-1}$)	pore volume ($cm^3\ g^{-1}$)	average pore size (nm)
75.61	0.13	7.34

Table S2 Relative atomic percentages of major elements on the MBC surface analyzed by EDS

Atomic %	C %	O %	Fe %
MBC	39.00	45.24	15.75

Table S3 Adsorption kinetics and isotherm parameters for NPs adsorption by MBC

The fitting parameters for the adsorption kinetics of NPs						
adsorbate	pseudo-first-order			pseudo-second-order		
	Q_e (mg g^{-1})	K_1 (h^{-1})	R^2	Q_e (mg g^{-1})	K_2 (mg g^{-1} h^{-1})	R^2
NPs-COOH	35.15	0.2	0.962	40.63	0.005	0.98
$NPs\text{-}NH_2$	73.49	0.72	0.872	82.42	0.013	0.941
adsorbate	Langmuir model			Freundlich model		
	Q_m (mg g^{-1})	K_L (L mg^{-1})	R^2	n	K_F (L mg^{-1})	R^2
NPs-COOH	39.87	0.19	0.783	1.94	7.07	0.923
$NPs\text{-}NH_2$	143.13	0.34	0.993	3	49.87	0.991

K_1 (h^{-1}), pseudo-first-order kinetic rate constant; K_2 (mg g^{-1} h^{-1}), pseudo-second-order kinetic rate constant; Q_e, (mg g^{-1}), equilibrium adsorption capacity; Q_m (mg g^{-1}), maximum adsorption; K_L (L mg^{-1}), distribution coefficient; K_F (L mg^{-1}), Freundlich constant; n, the favorability degree of the adsorption process.

Table S4 The mass recoveries of NPs at different experimental conditions under NaCl solution

	NPs	IS, mM	Column porosity	Recovery in column effluent (%)			
				M_{eff}	M_I	M_{II}	M_{Total}
Fig. 5 (a)	NPs-COOH	1	0.467	81.0	0.5	-	81.5
		5	0.467	22.6	19.2	9.2	51.0
Fig. 5 (b)	NPs-NH2	1	0.462	48.3	2.3	2.9	53.5
		5	0.468	-	19.8	10.6	30.5

IS, ionic strength; "-" denotes less than 0.01%; M_{eff}, M_I, and M_{II} are mass percentages of NPs recovered from column effluent in the transport phase and release phase I and phase II, respectively.

Table S5 The mass recoveries of NPs at different experimental conditions under NaCl solution

	NPs	IS, Mm	MBC, mg L^{-1}	Column porosity	Recovery in column effluent (%)			
					M_{eff}	M_I	M_{II}	M_{Total}
Fig. 6 (a)	NPs-COOH	1	0	0.467	81.0	0.5	-	81.5
			50	0.464	47.0	0.1	-	47.1
			100	0.469	42.5	1.3	3.9	47.6
Fig. 6 (b)	NPs-COOH	5	0	0.467	22.6	19.2	9.2	51.0
			50	0.463	4.1	30.5	11.4	46.0
			100	0.463	-	25.5	20.1	45.5
Fig. 6 (c)	NPs-NH_2	1	0	0.462	48.3	2.3	2.9	53.5
			50	0.463	41.4	3.6	3.2	48.3
			100	0.463	25.4	4.0	6.8	36.3
Fig. 6 (d)	NPs-NH_2	5	0	0.468	-	19.8	10.6	30.5
			50	0.463	-	35.3	12.9	48.1
			100	0.464	-	27.4	11.4	38.8

IS, ionic strength; "-" denotes less than 0.01%; M_{eff}, M_I, and M_{II} are mass percentages of NPs recovered from column effluent in the transport phase and release phase I and phase II, respectively.

Table S6 The mass recoveries of NPs at different experimental conditions under NaCl solution

	NPs	IS, Mm	MBC, mg L^{-1}	HA mg L^{-1}	Column porosity	Recovery in column effluent (%)			
						M_{eff}	M_I	M_{II}	M_{Total}
Fig. 7 (a)	NPs-COOH	5	0	0.0	0.467	22.6	19.2	9.2	51.0
				0.5	0.462	72.1	3.4	-	75.5
				1.0	0.463	86.7	0.7	-	87.4
Fig. 7 (b)	NPs-COOH	5	100	0.0	0.463	-	25.5	20.1	45.5
				0.5	0.463	26.3	8.9	2.2	37.5
				1.0	0.462	53.9	4.1	0.9	58.8
Fig. 7 (c)	NPs-NH_2	5	0	0.0	0.468	-	19.8	10.6	30.5
				0.5	0.463	74.1	3.9	-	78.0
				1.0	0.463	87.3	1.1	0.5	88.9
Fig. 7 (d)	NPs-NH_2	5	100	0.0	0.464	-	27.4	11.4	38.8
				0.5	0.463	38.5	10.1	2.1	50.7
				1.0	0.463	68.8	6.5	6.0	81.3

IS, ionic strength; "-" denotes less than 0.01%; M_{eff}, M_I, and M_{II} are mass percentages of NPs recovered from column effluent in the transport phase and release phase I and phase II, respectively.

Table S7 The mass recoveries of NPs at different experimental conditions under $CaCl_2$ solution

	IS	MBC	HA	Column	Recovery in column effluent (%)						
	Mm	mg L^{-1}	mg L^{-1}	porosity	M_{eff}	M_I	M_{II}	M_{III}	M_{IV}	M_V	M_{Total}
Fig. 8 (a)	1	0	0.0	0.464	28.9	0.2	-	0.4	-	-	29.5
	5	0	0.0	0.464	-	2.4	-	1.5	-	-	3.9
Fig. 8 (b)	1	0	0.0	0.464	28.9	0.2	-	0.4	-	-	29.5
		50	0.0	0.463	20.1	1.5	-	2.6	-	0.2	24.4
		100	0.0	0.463	9.0	1.0	-	0.5	-	0.4	10.9
Fig. 8 (c)	1	0	0.0	0.464	28.9	0.2	-	0.4	-	-	29.5
		0	0.5	0.463	53.3	-	-	-	-	0.4	53.7
		0	1.0	0.465	54.5	-	-	0.3	-	0.6	55.4
Fig. 8 (d)	1	0	0.0	0.464	16.7	0.3	-	2.3	-	2.8	22.0
	5	0	0.0	0.463	-	2.5	0.2	5.9	-	1.0	9.6
Fig. 8 (e)	1	0	0.0	0.464	16.7	0.3	-	2.3	-	2.8	22.0
		50	0.0	0.463	15.6	1.3	-	2.8	-	2.2	21.8
		100	0.0	0.462	10.6	1.8	0.1	3.5	-	3.6	19.6
Fig. 8 (f)	1	0	0.0	0.464	16.7	0.3	-	2.3	-	2.8	22.0
		0	0.5	0.463	27.4	-	-	1.2	-	2.5	31.1
		0	1.0	0.462	55.3	-	-	0.2	-	1.9	57.4

IS, ionic strength; "-" denotes less than 0.01%; M_{eff}, M_I-M_V are mass percentages of NPs recovered from column effluent in the transport phase and release phase I-V, respectively.

Table S8 Fitted values of NPs transport at different experimental conditions under NaCl solution

	NPs	IS, Mm	MBC, mg L^{-1}	HA, mg L^{-1}	k_1, min^{-1}	S_{max}/Co, cm^3 g^{-1}	R^2
Fig. 5 (a)	NPs-COOH	1	0	0	0.029	0.564	0.997
		5	0	0	0.328	0.813	0.937
Fig. 5 (b)	$NPs-NH_2$	1	0	0	0.098	1.866	0.995
		5	0	0	-	-	-
Fig. 6 (a)	NPs-COOH	1	0	0	0.029	0.564	0.997
		1	50	0	0.122	0.463	0.983
		1	100	0	0.256	1.284	0.994
Fig. 6 (b)	NPs-COOH	5	0	0	0.328	0.813	0.937
		5	50	0	0.522	1.435	0.957
		5	100	0	-	-	-
Fig. 6 (c)	$NPs-NH_2$	1	0	0	0.098	1.886	0.995
		1	50	0	0.125	1.564	0.999
		1	100	0	0.178	3.408	0.994
Fig. 6 (d)	$NPs-NH_2$	5	0	0	-	-	-
		5	50	0	-	-	-
		5	100	0	-	-	-
Fig. 7 (a)	NPs-COOH	5	0	0	0.328	0.813	0.937
		5	0	0.5	0.097	0.243	0.990
		5	0	1	0.032	0.136	0.995
Fig. 7 (b)	NPs-COOH	5	100	0	-	-	-
		5	100	0.5	0.216	1.137	0.979
		5	100	1	0.108	0.520	0.990
Fig. 7 (c)	$NPs-NH_2$	5	0	0	-	-	-
		5	0	0.5	0.055	0.263	0.992
		5	0	1	0.018	0.114	0.997
Fig. 7 (d)	$NPs-NH_2$	5	100	0	-	-	-
		5	100	0.5	0.153	0.946	0.981
		5	100	1	0.068	0.460	0.985

IS (mM), ionic strength of NaCl; *Co* (mg L^{-1}), input concentration of NPs; k_1 (min^{-1}), the first-order retention coefficient; S_{max}/Co (cm^3 g^{-1}), the normalized maximum solid-phase concentration of deposited NPs. R^2, Pearson's correlation coefficient.

Table S9 Fitted values of NPs transport at different experimental conditions under $CaCl_2$ solution

	NPs	IS, Mm	MBC, mg L-1	HA, mg L-1	k_1, min^{-1}	S_{max}/Co, cm^3 g^{-1}	R^2
Fig. 8 (a)	NPs-COOH	1	0	0.0	0.222	0.810	0.942
		5	0	0.0	-	-	-
Fig. 8 (b)	NPs-COOH	1	0	0.0	0.222	0.810	0.942
		1	50	0.0	0.278	1.140	0.980
		1	100	0.0	0.365	1.619	0.945
Fig. 8 (c)	NPs-COOH	1	0	0.0	0.222	0.810	0.942
		1	0	0.5	0.102	0.726	0.994
		1	0	1.0	0.120	0.509	0.968
Fig. 8 (d)	NPs-NH_2	1	0	0.0	0.314	1.243	0.984
		5	0	0.0	-	-	-
Fig. 8 (e)	NPs-NH_2	1	0	0.0	0.314	1.243	0.984
		1	50	0.0	0.294	1.585	0.980
		1	100	0.0	0.312	2.249	0.974
Fig. 8 (f)	NPs-NH_2	1	0	0.0	0.314	1.243	0.984
		1	0	0.5	0.211	0.898	0.933
		1	0	1.0	0.102	0.577	0.975

IS (mM), ionic strength of $CaCl_2$; *Co* (mg L^{-1}), input concentration of NPs; k_1 (min^{-1}), the first-order retention coefficient; S_{max}/Co (cm^3 g^{-1}), the normalized maximum solid-phase concentration of deposited NPs. R^2, Pearson's correlation coefficient.
